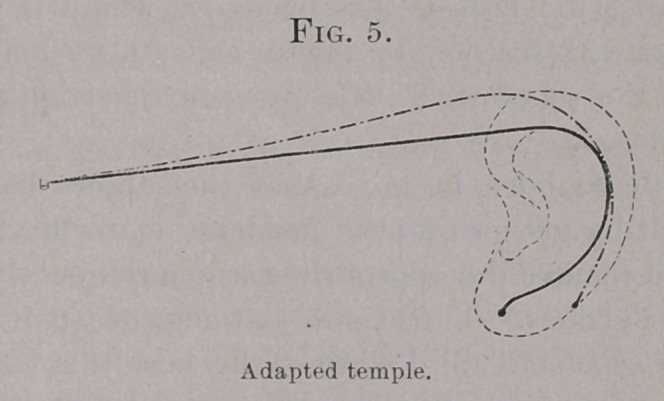# Philadelphia County Medical Society

**Published:** 1891-05

**Authors:** 


### PHILADELPHIA COUNTY MEDICAL SOCIETY.

*Stated Meeting, February 11, 1891.*

The President, John B. Roberts, M. D., in the chair.

Dr. Charles Hermon Thomas submitted a paper on

### THE CONSTRUCTION AND ADAPTATION OF SPECTACLE-FRAMES.

The treatment of ocular defects by means of glasses involves, beside the optical correction, a factor of no less practical importance—their mechanical adjustment. The purpose of the present paper is to direct attention to some of the mechanical aspects of the subject, particularly to the principles involved, and to certain methods of mounting spectacle-glasses.

The results of the most accurate refractive measurements may be entirely vitiated by a faulty position of the correcting glasses ; not only so, but new sources of eye-strain may be created by the very means adopted to remove an existing fault. Correcting glasses are remedial agents, just as orthopedic appliances are, and, as such, are powerful for evil as well as good, and hence everything belonging to them falls within the duty of the prescribing physician.

The optical centre of a lens is generally that part of the glass which we wish to bring before the pupil, as it and the part of the lens immediately surrounding it are freest from aberrations of all sorts—distort least. Occasionally, however, it may be desirable to displace this point by a definite amount ; in any case, we should insist on having the optician carry out our directions as regards the manner of mounting and the position of the glass with the same exactness that he employs in making it of the proper strength.

The purpose of the spectacle-frame is to hold a pair of glasses before the eyes in a definite position and with the least possible annoyance to the wearer. To accomplish this, I devised a plan about thirteen years ago (1878) for the construction of spectacle-bridges, which plan provides especially for a wide range of adaptability and the consequent accurate adaptation of spectacles to individual faces of almost every conceivable form. No account of the principles involved has heretofore been published, so far as is known, although some special forms of the bridge, as originally made under my direction, have come into almost universal use, being known throughout the optical trade under the name *saddle-bridge.*

Previous to the introduction of this bridge it was not practicable to obtain spectacle-frames suitable for persons with unusual forms of nose or face or with excessively prominent eyes or long lashes. Then, beside the ordinary "regular bridge," there was nothing better in use than the "X-bridge," or the equally unsatisfactory "snake-bridge," in both of which the combined weight of the glasses and frames was often borne directly upon the crest of the nose, besides which they usually failed to place the glasses in the correct position before the eyes. Few could wear either of the latter with comfort, and those who succeeded often did so only by padding them with wrappings of thread, thus making an unsightly cushion at the point of contact with the nose.

The bridge (Fig. 1) under the plan referred to consists of (1) a nose-piece of arched form, of flattened wire and made to conform

FIG. 1.

Saddle-bridge—typical form (back view).

accurately to the shape of the nose at a definite point of selection, crossing the bridge of the nose at right angles, and so resting saddle-wise upon it—whence its name. (2) A pair of adjustable return-pieces or arms, to the extremities of which are attached the rims or clasps carrying the glasses. These arms are produced by bending outward upon themselves the limbs of the wire from which the arch of the bridge has been formed, and are given whatever special direction may be required to place the glasses in the desired position before the eyes of the individual wearer.

The bridge consists, then, of an arch and two adjustable arms, which, while fixing the glasses in their proper position before the eyes, should furnish as nearly an immovable support as possible.

The bridge of the nose close to its root being the basis of support, the spectacle bridge must be constructed with reference to this part. The wire of which it is made should be wide at the middle and taper toward each end, so as to make the bridge widest where it takes its bearing on the sensitive part of the crest of the nose. Narrowing the extremities is of special advantage, as it facilitates any necessary bending at that point in the process of adjustment. The sides of the arch should embrace the nose snugly without undue pressure, and extend well back toward the inner canthus, but not far enough to press upon the lachrymal sac. The saddle, or arch, as thus described, becomes the fixed support when it rests in its proper position. This position varies considerably in different persons, though on every nose there is usually one best point which should be sought—the *point of selection*, it may be termed. Unless the arch be adjusted to this particular point, the wearer will be rendered uncomfortable, and be continually shifting his spectacles. A few days' wear may be required to determine this point definitely in a particular case.

The arch of the bridge, when once adapted to the nose, is not to be altered in position during any subsequent regulation or adjustment which may be required ; it is to be considered as a definitely fixed support, whose situation is determined, once for all, by the conformation of the wearer's nose. Hence, the position which the lenses are to take before the eyes does not directly depend upon the arch, but rather upon the length and direction of the adjustable arms attached to it, by variations in which the glasses may be made to take any required position. The arms are to be made long or short, they may be set high or low, pointed inward or outward, according to the requirements of any given case.

If the eyes be specially prominent, and the bridge of the nose be low, thus causing the lashes to project beyond the level of the nose, the arms must be made relatively long (Fig. 2); or if the bridge of

FIG. 2.

Saddle-bridge, with horizontal arms; for prominent eyes and long lashes.

the nose be low or flat, and the eyes be placed relatively high, it may be required to direct the arms perpendicularly upward (Fig. 3); or, again, if the bridge of the nose be prominent, and the eyes sunken, the arms should be shortened, or even reduced to the minimum required for purposes of lateral and vertical adjustment.

The height of the eye as related to the part of the nose on which the arch rests—the point of selection—determines the amount of

FIG. 3.

Saddle bridge, with vertical arms for flattened nose; eyes high.

slant, if any to be given to the arms. In practice it is found that in by far the larger proportion of cases the arms are nearly horizontal, slanting slightly upward; in exceptional cases they slant downward below the horizontal; and in rare instances it is necessary to give them an almost perpendicular direction upward. The angle which the arms make with the clamp or rim carrying the glasses must vary according to the direction of the arms, in order to keep the plane of the glasses perpendicular to the visual lines. The arm, where it is soldered to the rim, or the clasp of frameless glasses, is slightly bent in an upward direction. Increasing or diminishing these curves changes the position of the glasses vertically, and so compensates for any degree of upward or downward slant of the arms. This may be necessary where, for example, the point of selection of the arch is low down on the nose; the arms must then ascend vertically to raise the glasses to a level with the eyes; but this position of the arms will cause the glasses to assume an approximately horizontal direction—parallel to the visual lines—if the arms meet the rim at or about a right angle, as they usually do; in such a case, the arm must be bent so as to join the lens at oblique angle or even lie in the plane of the lens.

The proper adjustment of a pair of spectacles in ordinary cases is largely determined, as we have seen, by the length and direction of the arms. In special cases, also, as in asymmetry of the face, the compensation required is to be effected by the same means. In some cases the arms may need to be of unequal length. It is of frequent occurrence that the centres of the pupils on the two sides are unequally distant from the centre of the arch. When this condition exists, it is to be met by varying the direction and, it may be, also the length of the arms.

It is important from the point of view of the optician, to note that the principal adaptations of the bridge are preferably to be made extemporaneously and with the patient present. In this way, with a variety of sizes of the typical form at hand, the skilful mechanic is able to produce any particular modification which may be required without specially constructing the frames, even for atypical faces. It is often desirable to take the conformation of the nose at the point of selection. This may conveniently be done with lead wire, and the outline thus obtained may—by " rubbing "—be made a part of the record of the case.

Variations in the size of the lenses employed will also necessitate modifications in the lateral adjustment of the arms. To get advantage of a large glass in cases where the distance between the eyes is relatively small, the arms will have to be bent inward — made to approach each other. The opposite direction may have to be given them in cases of unusual width of face.

Lateral supports, or clamps, which take their bearing lengthwise on the sides of the nose near the base, as in eye-glasses of the best construction, have occasionally been employed by others in combination with spectacle-frames, but usually in form and by mechanical means not wholly satisfactory.

I have recently had made by the Fox Optical Company a combination of the eye-glass clamps with the saddle-bridge (Fig. 4), which

FIG. 4.

Saddle bridge; with clamps.

is neat and simple in construction, and which combines the advantages of both in great degree. The attachment is so made as to preserve the adjustability both of the bridge and the clamps. The special advantage of this combination is that it *distributes the pressure* over a larger surface, and upon parts better able to sustain it than does the arch of the bridge alone.

The side-pieces, or *temples*, should be *specially adapted* to the ear with as much care as the bridge is to the nose in each individual case. They should be hooked around the ears for constant use and be so formed as to retain the bridge at the point of selection on the nose, and thus secure a fixed position of the entire appliance. The curve of temples, as ordinarily made, is of far too great a radius. It takes its bearing behind the ear upon a limited surface, and so is liable to cut ; it fails to secure a proper hold to prevent its riding upward, and it often exerts spring-pressure productive of pain and injurious to ears and nose alike.

An adapted temple, designed to fulfil the above indications and obviate these defects, has recently been constructed under my direction, and has been the test of use so well as to justify its continued regular employment (Fig. 5). The wire of which it is made passes

FIG. 5.

Adapted temple.

back in a straight line to the top of the ear, at which point it is bent somewhat abruptly downward, and is made to conform accurately to the posterior surface of the conch close to its junction with the head, where it rests in contact with the ear, but without perceptible pressure. Asymmetry in the height of the ears, causing *tilting of the frames from the level*, is to be met by a compensating adjustment in the temples, *i. e.*, bending the temple upward on the side of the higher or downward on the side of the lower ear—or both —and so dividing the result between the two sides. The glasses should be slightly inclined from the perpendicular, so as to bring the lower edges somewhat nearer the face than the upper, which is to be effected by giving the temples the appropriate angulation at their junction with the hinges when it is impracticable to change the direction of the hinges themselves.

The material of the frames should usually be gold, of a good quality and of a weight as light as is consistent with strength and steadiness. Steel rusts too readily and is not well adapted to the adjustments frequently required—more especially in the temples. Silver is so soft as to be almost worthless. The lenses themselves should usually be as large as the face of the wearer permits : seldom less than 28x38 mm. for an adult, and not infrequently as large as 29x40 or 30x42 mm., in order that the eyes may be well covered in their ordinary lateral movements. Such large lenses are hardly more conspicuous than small ones—especially if frameless glasses be used—because they allow the eye itself to be easily seen. The reflections from the edges of frameless glasses which are so annoying to some persons may be avoided by slightly dulling the polish on the lower edge; the source of this reflected light being usually at or above the level of the eyes, the reflection enters the eye from this edge alone.

The glasses should be worn as close to the eyes as possible, without touching the lashes. Occasionally, where the lashes are especially long, with feathery or uneven ends, they should be neatly trimmed with the scissors—a little procedure best practised when the eyes are closed.

It is also to be born in mind that the subject has an artistic aspect and that by giving proper consideration to this phase much can be done to remove the opprobrium which frequently attaches to the wearing of glasses. The neat adjustment of a pair of frameless gold-mounted spectacles is doubtless the best that can be accomplished with spectacles in this respect.

In the above it will be seen I have limited myself to a description of no one form of bridge, nor even of a number of special forms, but the effort has been made rather to demonstrate *the mechanical principles involved in the construction and adaptation of spectacle-frames suitable to all the requirements of practice.* By the means proposed it is practicable to secure the correct position of the glasses before the eyes, together with comfort to the wearer and a satisfactory artistic effect, thus fulfilling the three principal indications of spectacle-mounting.

### DISCUSSION.

Dr. EDWARD JACKSON: One of the difficulties I have met with in having opticians fit frames, and in making students understand how frames should fit, is in regard to the location of this "point of selection," to which Dr. Thomas refers. It is not any point arbitrarily chosen, but is, in each case, rigidly determined by the form of the face. To it the traction of the temples constantly tends to bring the bridge.

The bridge placed above it is drawn down, or below it is drawn up toward it and comes to rest upon it.

A point which has recently come to my notice in fitting frames is that the plane of the temples must pass through that part of the surface of the bridge that bears upon the nose. If it passes above or below this it tends to tilt the bridge, so that its edge bears on the nose instead of the flat surface. To effect the proper position it will sometimes be needful to attach the bridge and the joint for the temple, not at opposite extremities of the horizontal diameter of the lens ellipse, but at the extremities of a shorter cord lying above or below this diameter.

Dr. George M. Gould: I wish to speak on one point brought up by Dr. Thomas, and that is the reflection from the edge of rimless glasses. I have had patients who could not wear glasses on account of the annoyance caused by this reflection. Last year, in *Knapp's Archives*, I described a little device of my friend Dr. Rhoads, by which the edge of the glass was beveled on a plane with the pupil. In this way all reflection is avoided. The only objection is that this exaggerates the reflection to the beholder.

In reference to the effects of pressure of the bridge on the nose, I had a case last week which brought a new phase of this matter before me. A couple of months ago I applied glasses to a patient with specific rhinitis. Following this, the nose ulcerated near the point of pressure, and several pieces of bone were discharged. I do not think that it was due altogether to misfitting of the frame, but principally to the fact that the skin was so sensitive that the least pressure caused trouble. It, however, gave me a lesson not to apply glasses in specific rhinitis in an acute stage.

The whole of the paper of Dr. Thomas is a corollary to the great fact that the optician should be an educated mechanic. The optician stands in the same relation to the oculist that the druggist stands to the physician. Until the optician learns to take a pride in his profession we shall not have well-fitted glasses, unless we are constantly on the watch. We should, therefore, do all that we can to elevate and encourage the dignity of the optician's profession.

Dr. Thomas: I am glad to hear the suggestions of Dr. Jackson in regard to the line of draught and the location of the temples. I think that there are cases in which this may make a good deal of difference, and it is a point which hitherto I have not taken into account.

The bridge has had a widely extended use, for a number of years, and the only reason for bringing the subject forward now is that it is not perfectly understood by ophthalmologists and opticians. It is a bridge of wide adaptability, and is capable of being converted into a great variety of special forms, some of which have been here shown.

## Figures and Tables

**Fig. 1. f1:**
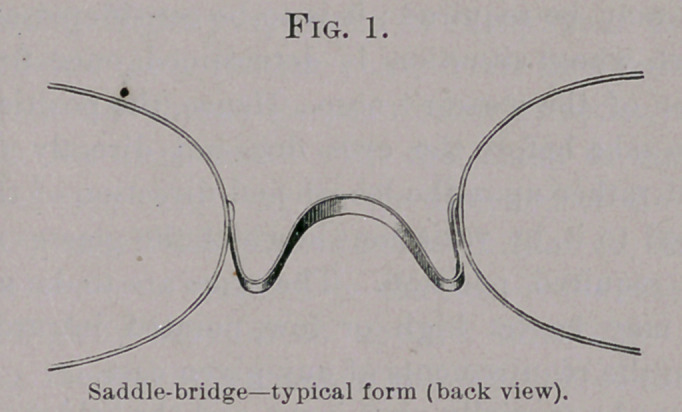


**Fig. 2. f2:**
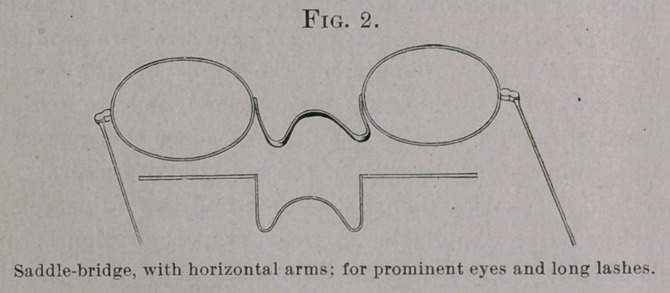


**Fig. 3. f3:**
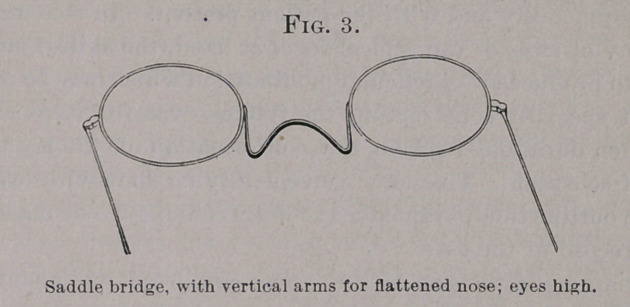


**Fig. 4. f4:**
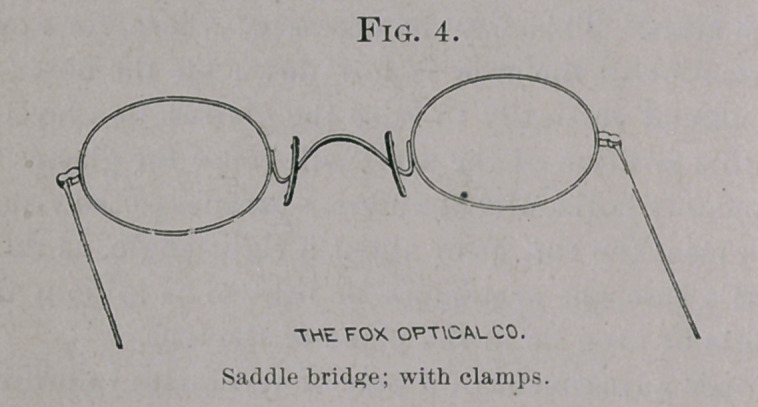


**Fig. 5. f5:**